# Focal Nodular Hyperplasia and Hepatocellular Adenoma around the World Viewed through the Scope of the Immunopathological Classification

**DOI:** 10.1155/2013/268625

**Published:** 2013-04-14

**Authors:** Charles Balabaud, Wesal R. Al-Rabih, Pei-Jer Chen, Kimberley Evason, Linda Ferrell, Juan C. Hernandez-Prera, Shiu-Feng Huang, Thomas Longerich, Young Nyun Park, Alberto Quaglia, Peter Schirmacher, Christine Sempoux, Swan N. Thung, Michael Torbenson, Aileen Wee, Matthew M. Yeh, Shiou-Hwei Yeh, Brigitte Le Bail, Jessica Zucman-Rossi, Paulette Bioulac-Sage

**Affiliations:** ^1^Inserm U1053, Université Bordeaux Segalen, 33076 Bordeaux Cedex, France; ^2^Institute of Liver Studies, King's College Hospital, London, UK; ^3^National Taiwan University College of Medicine, Taipei, Taiwan; ^4^National Taiwan University Hospital, Taipei, Taiwan; ^5^Department of Pathology, University of California, San Francisco, CA 94143-0102, USA; ^6^Department of Pathology, Mount Sinai School of Medicine, New York, NY 10029, USA; ^7^Institute of Pathology, University Hospital, 69120 Heidelberg, Germany; ^8^Department of Pathology, Yonsei University College of Medicine, P.O. Box 8044, Seoul, Republic of Korea; ^9^Service d'Anatomie Pathologique, Cliniques Universitaires Saint Luc, Université Catholique de Louvain, 1200 Brussels, Belgium; ^10^Department of Pathology, Johns Hopkins University School of Medicine, Baltimore, MD, USA; ^11^Department of Pathology, Yong Loo Lin School of Medicine, National University of Singapore, National University Hospital, National University Health System, Singapore 119074; ^12^Department of Pathology, University of Washington School of Medicine, Seattle, WA, USA; ^13^Pathology Department, Hôpital Pellegrin, CHU Bordeaux, 33076 Bordeaux Cedex, France; ^14^Inserm, UMR-674, Génomique Fonctionnelle des Tumeurs Solides, IUH, 75010 Paris, France; ^15^Université Paris Descartes, Labex Immunooncology, Sorbonne Paris Cité, Faculté de Médecine, 75005 Paris, France

## Abstract

Focal nodular hyperplasia (FNH) and hepatocellular adenoma (HCA) are benign hepatocellular tumors. The risk of bleeding and malignant transformation of HCA are strong arguments to differentiate HCA from FNH. Despite great progress that has been made in the differential radiological diagnosis of the 2 types of nodules, liver biopsy is sometimes necessary to separate the 2 entities. Identification of HCA subtypes using immunohistochemical techniques, namely, *HNF1A*-inactivated HCA (35–40%), inflammatory HCA (IHCA), and beta-catenin-mutated inflammatory HCA (b-IHCA) (50–55%), beta-catenin-activated HCA (5–10%), and unclassified HCA (10%) has greatly improved the diagnostic accuracy of benign hepatocellular nodules. If HCA malignant transformation occurs in all HCA subgroups, the risk is by far the highest in the **β**-catenin-mutated subgroups (b-HCA, b-IHCA). In the coming decade the management of HCA will be more dependent on the identification of HCA subtypes, particularly for smaller nodules (<5 cm) in terms of imaging, follow-up, and resection.

## 1. Introduction

The knowledge of benign hepatocellular tumors, that is, focal nodular hyperplasia (FNH) and hepatocellular adenoma (HCA), has considerably progressed in the last 10 years, thanks to molecular biology, followed by immunohistochemical applications. Following these advances, new classification is now largely used, first in France, and more recently in other European, American, and East countries.

The aims of this study are (1) to make a brief general overview of these 2 entities [2–8]; (2) to report results of a survey through different academic centers in France and throughout the world; (3) to report applications of the molecular/immunohistochemical data and of the new HCA classification in practice through the Bordeaux experience.

## 2. A Brief Overview of Focal Nodular Hyperplasia and Hepatocellular Adenoma

### 2.1. Focal Nodular Hyperplasia

It is the second most frequent benign liver nodule (after hemangioma), occurring in 0.8% of an adult autopsy population and has been reported in 0.6–3% of the general population. In 80–90% of cases, FNH is discovered in women in their third or fourth decade. In countries (i.e., China) where OC use has been less prevalent, FNH tends to be a lesion of adult men or children of either gender.

FNH is solitary in 2/3 of cases. Most lesions are asymptomatic and are therefore discovered as incidental findings during surgery, autopsy, or imaging procedures for unrelated symptoms. Large lesions can present with abdominal pain or compression of adjacent organs. Reports of hemorrhage or malignant transformation probably do not exist or are exceptional and require confirmation. FNH lesions may regress with age as shown by disappearance of presumed FNH lesions on serial imaging studies. 

The background liver is usually normal. FNH is associated with hepatic hemangioma in 20% of cases. Coexistence with HCA is not rare. Associated lesions outside the liver have been reported such as hemihypertrophy, hemangioma of cervix, vascular malformations of brain, and meningioma.

#### 2.1.1. Typical FNH: Morphological Features

*(1) Gross Findings*. On cut section, classical FNH is a pale, firm mass measuring from a few millimeters to more than 10 centimeters in diameter. The margin is well delimited, and the mass is lobulated and non encapsulated. The lesion is composed of nodules each measuring 2-3 mm, separated by zones of atrophy that give the lesion a multinodular appearance. The lesion characteristically has a central or eccentric stellate fibrous scar with radiating extensions that partially surround some component nodules.

*(2) Microscopic Findings.* FNH lesions are composed of nodules of benign-appearing hepatocytes arranged in plates not more than 2 cells in thickness. Steatosis may occur, usually focal. The central scar is often edematous or congested and contains one or more large dystrophic vessels, accompanied by numerous small arterioles. The large vessels have irregular fibrous thickening of the intima with focal thinning of the media. The internal elastic lamina is poorly formed and reduplicated. The portal vein is absent. The central fibrous region has radiating branches composed of portal tract-like structures that contain an artery unaccompanied by portal veins or ducts. When fibrous septation is prominent, the appearance may be indistinguishable from cirrhosis, especially in biopsy specimens. A lymphocytic or mixed inflammatory infiltrate is frequent in fibrous regions. At the interface between fibrous regions and nodules, there are often features of cholate stasis including feathery degeneration of hepatocytes, Mallory-Denk bodies, and a ductular reaction that may be highlighted with CK7 and CK19 immunostaining. Sinusoids adjacent to arterial sources are lined by CD34-positive endothelium. Glutamine synthetase is a very useful immunostain, showing a characteristic broadband of expression in hepatocytes often near the hepatic veins.

#### 2.1.2. Atypical FNH

Incomplete or early forms may lack a central scar; they have an incompletion (or absence) of multinodular organization and sometimes exhibit more or less prominent regions of congestion.

#### 2.1.3. Molecular Features

Clonal analysis using the HUMARA test demonstrated the reactive polyclonal nature of liver cells in FNH in 50–100% of the cases [2, 7]. Messenger RNA (mRNA) expression levels of the angiopoietin genes (ANGPT1 and ANGPT2) involved in vessel maturation are altered, with the ANGPT1/ANGPT2 ratio increased compared with normal liver, cirrhosis, and other liver tumors. These data support the importance of vascular alterations in the pathogenesis of FNH. The beta-catenin pathway is activated, including the downstream target, glutamine synthetase [9]. This activation explains the expansion of hepatocytes expressing glutamine synthetase that is so useful for histologic diagnosis. The molecular mechanisms of this activation are uncertain, but do not involve demonstrable mutations in beta-catenin or Axin1.

The pathogenesis of FNH is not fully established. The association with conditions having local or systemic vascular anomalies and the presence of unusually large vessels within the lesions has led to the belief that FNH is a nonspecific response to focally increased blood flow.

### 2.2. Hepatocellular Adenoma

 It is a rare benign liver neoplasm composed of hepatocytes. The incidence is around 3-4/100 000 in Europe and North America and is lower in Asia. 85% of cases occur in young women; HCA is rare in children, men, and the elderly.

The major risk factor for development of HCA is the exposure to estrogenic or androgenic steroids. In young women, 80% have been users of oral contraceptives (OC). The risk increases with the duration and type of OC usage. The prevalence appears to be declining as low-estrogen preparations have become more widely used. The lesions usually decrease in size after stopping OC or after menopause. The clinical presentation of HCA may include abdominal pain, abdominal mass, intraperitoneal hemorrhage, abnormal liver tests, or space occupying lesion found incidentally on an imaging study. HCA can be single or multiple. When 10 or more adenomas occur, the condition is known as adenomatosis. Clinically significant hemorrhage is observed in 20–25% of cases; the risk is highest when the tumors are larger than 5 cm. Malignant transformation to hepatocellular carcinoma (HCC) is rare, but well documented, occurring in up to 7% of cases as reported from referral centers. The risk of transformation varies with the HCA subtype (see below) and with the clinical association, being higher in patients with glycogenosis or androgenic-anabolic steroid use.

#### 2.2.1. General Morphological Data

*(1) Gross Findings.* Hepatocellular adenomas are typically large globular tumors with prominent vessels in the overlying hepatic capsule. On cut section, the tumor parenchyma is soft and relatively uniform although areas of congestion, necrosis, hemorrhage, or fibrosis are frequent. The margins of the lesion are ill-defined both grossly and microscopically, with little or no fibrous capsule. Lesions vary in size from microscopic up to 20 cm in diameter. In livers with adenomatosis, there may be hundreds of lesions visible as minute ill-defined nodules visible grossly or only microscopically. HCA may be similar in color and texture to the background liver but are more easily seen when there is lesional steatosis, major congestion and hemorrhage or degenerative changes. The background liver is usually normal, though there may be pallor, fibrosis, or brown pigmentation related respectively to fatty liver disease, glycogen storage disease, iron overload, or other diseases.

*(2) Microscopic Findings*. HCA is typically composed of benign hepatocytes arranged in regular liver cell plates that are usually one, or at most two, cells in width. A pseudoglandular growth pattern may be seen focally. Tumor hepatocytes have cytoplasm that may be normal, clear (glycogen-rich), steatotic, or contain pigment in lysosomes. Nuclear atypia and mitoses are unusual. The tumor parenchyma is supplied by isolated arteries unaccompanied by bile ducts. Variations in this typical pattern are frequently seen in some of the subtypes, as described below. 

#### 2.2.2. Molecular Features

The reader is referred to the paper by Nault et al. in this issue. Briefly,

*(1) HNF1*α*-Inactivated HCA (H-HCA).* The *HNF1A* gene encodes the hepatocyte nuclear factor 1 (HNF1*α*), a transcription factor that is involved in hepatocyte differentiation. Bi-allelic inactivating mutations of this gene is found in approximately 35–40% of HCA; 90% of *HNF1A* mutations are somatic; in 10%, they are constitutional (germline). Heterozygous germline mutations in *HNF1A* are responsible for an autosomal dominant form of diabetes MODY3 (maturity onset diabetes of the young type 3). In patients with MODY 3 and HCA, there is an additional somatic mutation of the second allele in the tumor.

*(2) Beta-Catenin Activated HCA (b-HCA).* An activating *β*-catenin mutation is found in 10–15% of HCA cases. Glul, a target gene of *β*-catenin, coding for the protein glutamine synthetase (GS) is also upregulated.

*(3) Inflammatory HCA.* Inflammatory HCA (IHCA), represent more than half of HCA cases. They are characterized by increased expression of inflammation-associated proteins such as serum amyloid A (SAA) and C-reactive protein (CRP), at both the mRNA and protein levels. 60% of these adenomas harbor mutations in gp130. Mutant gp130 activates STAT3 in the absence of its ligand, which is IL-6. Beta-catenin mutations may coexist with gp130 mutations in 10% of IHCA.

*(4) Unclassified HCA.* HCA without distinguishing histological features and without known mutations represent less than 10% of all cases. 

### 2.3. Diagnosis of FNH and HCA

An accurate diagnosis can be made using imaging techniques in 90% of cases in experienced centers. Contrast enhanced ultrasonography (CEUS) is the first modality of choice for FNH. MRI is the first modality of choice for HCA. In less than 10% of cases, the differential diagnosis of FNH, HCA, and hepatocellular carcinoma (HCC) cannot be solved by imaging alone. A biopsy interpreted by an experienced liver pathologist can resolve most of these problem cases, with standard and/or immunohistochemical stainings. If the biopsy is not definitive, surgery may be advocated.

The first task is to be certain that the biopsy includes the lesion. Therefore, it is recommended that a biopsy be accompanied by a sample of non-lesional liver. 

### 2.4. Differential Diagnosis

HCA is the most frequent lesion to be distinguished from FNH. The histologic diagnosis of FNH requires two main criteria. The lesion must be composed of benign-appearing hepatocytes and must be supplied by altered portal tracts. Adenomas are supplied by isolated arteries, not portal tracts. A source of difficulty, in this regard, is the presence of CK7/CK19-positive ductular elements in HCA of the inflammatory type. The key feature is that glutamine synthetase expression shows a distinctive map-like distribution adjacent to hepatic veins in FNH while expression is diffusely positive in beta-catenin-activated HCA and mostly negative in other types of HCA (see the following).

Macroregenerative nodule/FNH-like in cirrhotic patients and patients with vascular alterations may be difficult to differentiate from FNH or HCA. These nodules share some similarities with FNH but differ in some ways: the beta-catenin pathway is not activated in cirrhotic FNH-like nodules [9]; the ANGPT1/ANGPT2 ratio is not increased; GS is absent or mildly expressed; inflammatory proteins such as CRP may occasionally be expressed. The presence of focal hepatic vein obstruction and the association with Budd-Chiari syndrome suggest a role of outflow obstruction.

HCC may mimic FNH including focal scarring, arterialized sinusoids, and residual portal tract remnants. Warning signs of malignancy include nuclear pleomorphism, high N/C ratio, wide plates, and mitotic figures in the lesion and often cirrhosis in the background liver. 

The differential diagnosis between HCA and well-differentiated HCC remains difficult.

## 3. HCA/FNH throughout the World

At the end of the last century it was thought that HCA will disappear with the use of OC of the third generation. This was not the case. There are several reasons for that: the wider use of modern imaging techniques, the better identification of HCA among hepatocellular tumors, and the emergence of new etiological factors such as obesity [10, 11]. HCA still remains a challenge for clinicians, radiologists, and pathologists. The number of publications is still rising (a pubmed search using hepatocellular adenoma as criterion in 1980–1984, 1990–1994, 2000–2004, and 2005–2009 gave 28, 30, 69, and 87 publications, resp.). Surgery (or any other method to eliminate the HCA) is still necessary to prevent the risk of hemorrhage which can be lethal and the HCC transformation. These risks being absent in FNH, surgery is not recommended; surgery is, however, still performed. There are several reasons for that: surrounding organ compression, compression of liver vessels and biliary tree, pain, and perhaps more importantly doubt about the nature of the tumor. From a brief survey performed in some academic centers (Table 1), as well as from other publications reported in this issue or elsewhere, it is obvious that surgery and biopsies are still performed in France, Europe, and the US for FNH and HCA. The percentage of the different HCA subtypes are in the range previously published (Table 1). The great difference concerns Asia where HCA is extremely rare [12, 13] probably because of other means of contraception than oral contraceptives. 

Today the use of the HCA immunohistochemical classification [1, 14–19] is spreading. Several centers have published their own data [20–24].

## 4. The Diagnosis of HCA Subtypes in Routine Practice: The Bordeaux Experience

The pathological diagnosis of benign liver tumors should take into account the clinical, biological [25], and radiological data [26–35] including etiology.

**Figure 1:**
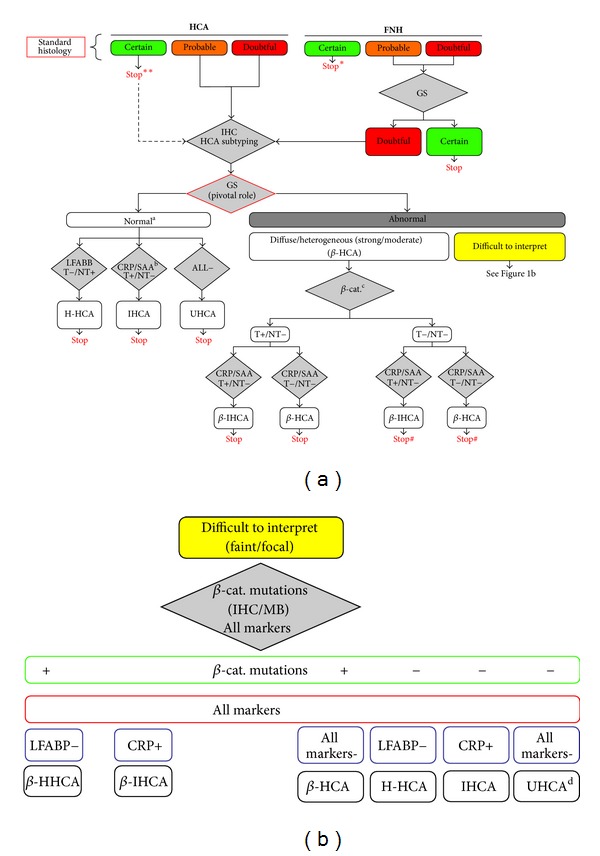
Adapted from Bioulac-Sage et al., [1]. Algorithm for immunohistochemical (IHC) diagnosis of benign hepatocellular nodules: focal nodular hyperplasia (FNH) and hepatocellular adenoma (HCA). Glutamine synthetase (GS) is not always mandatory for the diagnosis of *FNH or **HCA in routine practice. IHC is mandatory for HCA subtyping: markers are presented in grey square with their results positive (+) or negative (−) in tumor (T) and nontumoral liver (NT). LFABP: liver fatty acid binding protein; CRP: C-reactive-protein. Final diagnosis of HCA subtypes is: HNF1a inactivated (H-HCA), inflammatory (IHCA), B-catenin activated (B-HCA), B-catenin activated inflammatory (B-IHCA), or unclassified (UHCA). ^a^: GS negativity or positivity limited at the periphery and/or around some veins within HCA. ^b^: serum amyloid A staining is usually less sensitive and specific than CRP. ^c^: aberrant B-catenin nuclear staining. ^#^: needs molecular biology confirmation. ^d^: can be difficult to differentiate from FNH.

Main informations are summarized in Box 1. The gross anatomy (nodule and nontumoral liver) remains an essential part of the diagnosis and is essential for the sampling. We recommend to take pictures of the sampled areas and to sample all areas that look different. In the non tumoral liver, it is recommended to sample even tiny areas that look abnormal. To facilitate IHC interpretation, we sample areas at the junction of tumor and nontumoral tissue. The histological features are recorded to classify HCA (Box 2). The IHC data observed in benign liver nodules are summarized in Table 2; to reach a diagnosis, not all IHC techniques are used. Markers are used according to the algorithm in Figure 1.

Briefly *H-HCA* represents a homogeneous group of tumors with lobulated contours, showing typically marked and diffuse steatosis, absence of significant inflammation, or nuclear atypia. FABP1, coding for L-FABP (liver fatty acid binding protein), is a gene positively regulated by *HNF1A*, expressed in normal liver tissue and clearly downregulated in this HCA subtype. By immunohistochemistry, there is a nearly complete absence of LFABP staining, contrasting with the nontumoral surrounding liver which appeared homogeneously stained (even though faintly). Therefore, lack of LFABP expression is a very good diagnostic argument for HNF1-alpha-inactivated HCA, specific of this subtype, since there is a very good concordance between this immunophenotype and *HNF1A* mutations. Furthermore, the downregulation of LFABP may contribute to the fatty phenotype through impaired fatty acid trafficking. HNF1A-mutated HCA occurs almost exclusively in women. Nodules can be solitary or multiple. Most of adenomatosis is *HNF1A* mutated. Constitutional mutations can affect both sexes and can be discovered in children, sometimes as a familial form or associated with MODY 3.

*B-HCA.* This HCA subtype is often associated with specific conditions (i.e., glycogenosis, male hormone administration) and male gender. The lesions are usually solitary (except in glycogenosis) and have an increased risk of malignant transformation, compared to the other subtypes. Steatosis and inflammation are usually absent. Nuclear atypia and a pseudoglandular growth pattern are frequent in this subtype, so that distinction from well-differentiated HCC may be very difficult. By immunohistochemistry, glutamine synthetase is usually strongly expressed in a diffuse pattern, associated with aberrant cytoplasmic and nuclear expression of beta-catenin. When glutamine synthetase staining is heterogeneous and beta-catenin nonconclusive, molecular biology remains the method of choice for identifying beta-catenin mutation.

*IHCA.* Most patients with IHCA are women. Obesity and fatty liver diseases are frequent. In 50% of cases there are elevated serum levels of CRP and increased erythrocyte sedimentation rate, rarely in association with fever and anemia, features which can regress after HCA resection. Nodules can be solitary or multiple. Micronodules can also be detected by SAA and CRP immunostaining in the liver parenchyma, outside the main tumors. Histologically, IHCA typically exhibits focal or diffuse inflammation, sinusoidal dilatation, congestion and peliotic areas, and numerous and thick-walled arteries often associated with ductular reaction, lying in small amount of connective tissues. Steatosis may be seen, mainly focally. Immunohistochemistry demonstrates strong expression of SAA and CRP restricted to tumoral hepatocytes. There is a risk of malignant transformation, particularly for IHCA which is also beta-catenin mutated.

Reasons for classifying HCA are summarized in Box 3.

Major advances brought by the classification are (a) individualization of FNH from HCA and FNH from FNH-like, (b) identification of MODY3 and patients at high risk of malignant transformation, (c) demonstration that adenomatosis was not per se a specific HCA subtype, but an entity defined by an arbitrary number of nodules > ten detected by imaging techniques (the high number linked probably to a specific susceptibility), and (d) correction of errors (the so-called telangiectatic FNH being IHCA).

**Figure 2:**
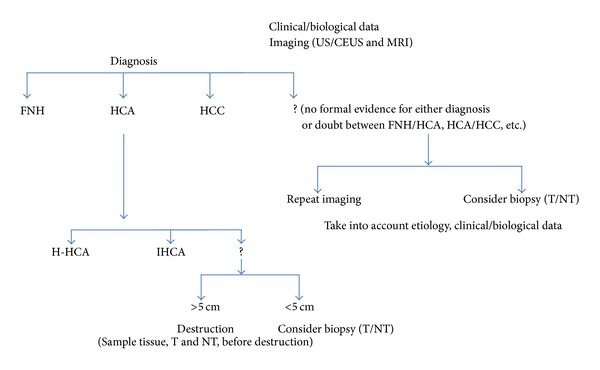
Practical guidelines for the identification of HCA subtypes (outside the emergency context).

Practical guidelines for the diagnosis of HCA subtypes are summarized in Figure 2.

The HCA classification has changed the way we consider HCA and FNH in our center. The diagnosis accuracy of FNH and HCA has completely changed at the end of the first 2000 decade. There are probably in the literature many unavoidable diagnostic errors concerning benign hepatocellular nodules particularly in the field of hemorrhagic FNH, malignant transformation of FNH, identification of difficult nodules, and so forth. 

### 4.1. Practical Guidelines for the Management of HCA Subtypes [25, 36–56]

For practical reasons once the diagnosis of liver tumors is made, the patient is often referred to a surgeon. In spite of the vast literature in various domains: imaging, pathology, surgery, complications, and so forth, there is no guidelines for the diagnosis and treatment of HCA. Nevertheless the major contribution of some European centers (see Supplemental Table 1 in Supplementary Material available online at http://dx.doi.org/10.1155/2013/268625) is a good starting point to obtain valuable information.

There is a global consensus among liver surgeons that adenoma >5 cm should be resected if they have not regressed after stopping oral contraceptives. Some surgeons prefer that all HCA should be removed particularly if they are easily accessible laparoscopicaly. Indeed, the clinical presentation in terms of age, sex, oral contraceptives and other major etiology, number of nodules (from single, to several and up to myriads) and size (2 to 20 cm), mode of discovery, and so forth, therefore it is extremely difficult to define acceptable guidelines based on solid arguments. Most of the series published are very small and it is difficult to make a judgment on case report. 

This is the reason why we believe that to make progress we need to collect prospective data from different countries and continents. The identification of subtypes is one of the key factors among others that need to be collected (Box 1). The ideal situation would be to follow strictly well- identified (MRI/biopsy) HCA particularly those at a high risk of malignant transformation (*β*-HCA and *β*-IHCA).

**Figure 3:**
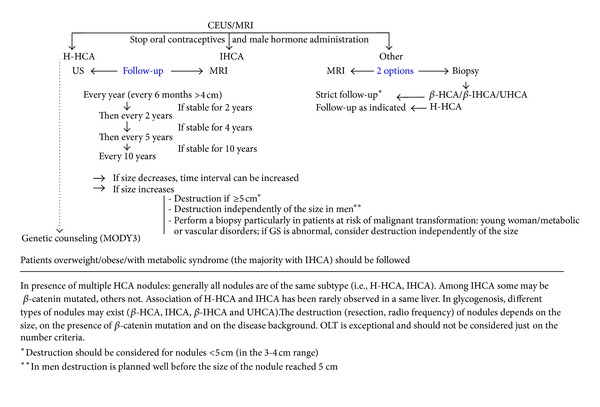
Management of HCA subtypes <5 cm.

In the absence of accepted guidelines, our proposition for the management of HCA subtypes <5 cm is summarized in Figure 3. 

### 4.2. Future Developments

It is clear that we have entered a new era in the study of benign hepatocellular tumors and perhaps more importantly in the field of HCC developing in nonfibrotic liver. One may still consider that this breach has not yet dramatically changed our strategies—diagnostic and therapeutic [38]. This is only partly true (see above) because it will take time before the classification brings its full potential. It is true that our knowledge is still limited. Future development will be based on imaging techniques [35], molecular data including chromosomal abnormalities [57], on the ability to combine molecular, radiological, and clinical data, and first of all on the reinterpretation of the routine histopathologic and IHC data considering the new molecular data (work in progress). 

Because HCA is rare, it is necessary to combine data from different centers. The main task is to better define patients at risk of malignant transformation [58, 59], to understand the susceptibility of some individuals, compared to controls, to develop nodules, either one while others develop myriads, the susceptibility of some nodules to grow while others remain undetectable, to transform while others remain quiescent, and to regress or not. Other main objectives are to study on a large scale HCA in various etiological conditions particularly in vascular diseases and in different liver diseases with different pathological backgrounds particularly NASH and cirrhosis [60].

Deciphering the molecular pathways leading to nodule formation, one can imagine that the medical treatment of HCA is not out of hand.

## 5. Conclusion

The diagnosis and prognosis of benign hepatocellular nodules have changed in the recent years. This will have an impact on the management of these nodules including surveillance. 

## Supplementary Material

Supplementary Table 1: main publications from European centers dealing with hepatocellular nodules.

## Figures and Tables

**Box 1:**
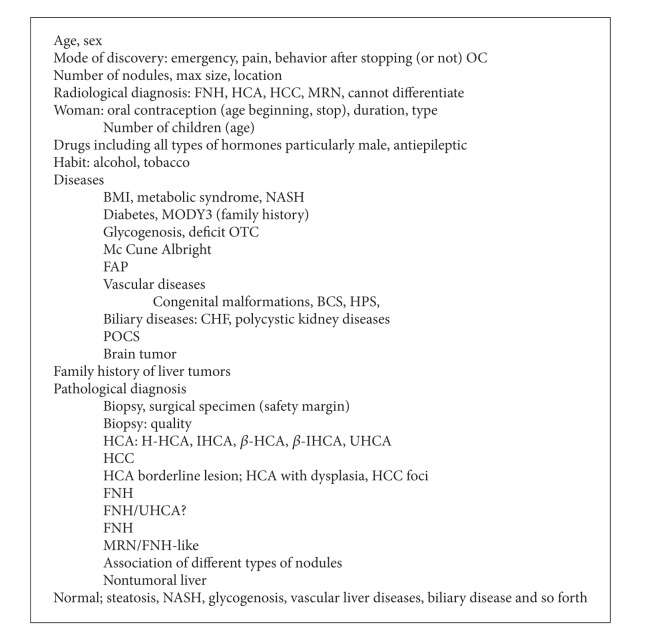
Clinical and pathological information useful to manage the patient.

**Box 2:**
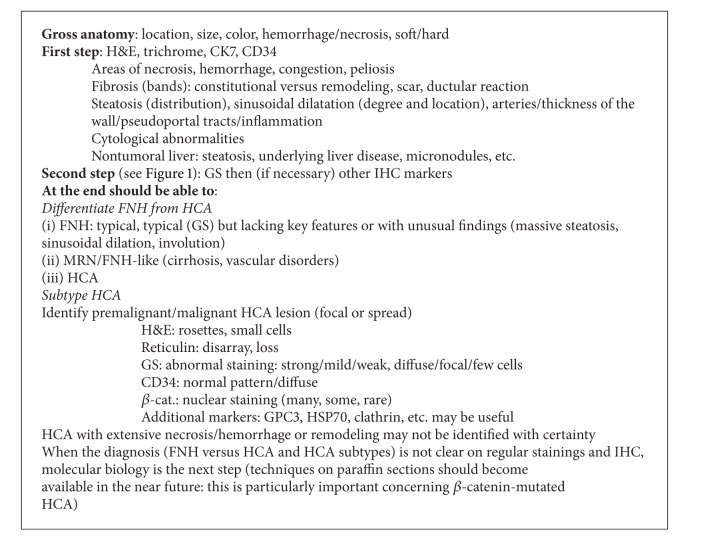
Pathological record.

**Box 3:**
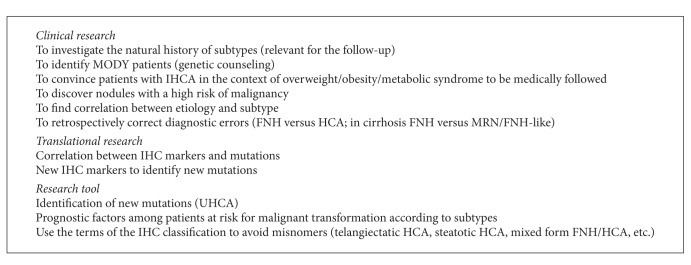
Reasons to classify hepatocellular nodules using IHC methods.

**Table 1:** Diagnosis of FNH and HCA performed in different academic centers.

	HCA		FNH		No final diagnosis	
	Surgery	Biopsy	Surgery	Biopsy	Surgery	Biopsy
French (from 2008 to 2011)						
(1) Besançon	4 (4♀)	10	13	3	0	0
(2) Bordeaux	49 (44♀)	16	27	29	0	2
(3) Caen	22 (20♀)	6	19	6	0	0
(4) Créteil	14 (13♀) 2 with HCC	19 3 with HCC	19	13	0	0
(5) Grenoble	19 (18♀)	18	3	12	0	1
(6) Lille	31 (26♀) 1 with HCC	30	25	39	2	6
(7) Lyon (1)	49 (41♀) 4 with HCC	13	17	16	4	7 (1 with HCC)
(8) Lyon (2)	16 (13♀) 1 with HCC	25	14	22	0	4
(9) Montpellier	32 (31♀) 1 with HCC	28	15	25	3	1
(10) Nice	32 (31♀) 1 with HCC	28	3	25	3	1
(11) Paris (St. Antoine)	11 (9♀) 1 with HCC	13	20	14	0	2 (1 with HCC)
(12) Villejuif (Gustave Roussy)	1 (1♀)	3	5 (4♀)	6	0	4

International						

(1) Baltimore (from 1984 to 2012)	63 (61♀) 7 with HCC	6	79	54	4	8
(2) Brussels (Cliniques universitaires Saint-Luc-UCL) (1992–2012)*	37 (33♀): 21 IHCA, 10 H-HCA, 1 with IHCA + H-HCA, 3 *β*-HCA (2 with HCC), 2 UHCA	14	22	14		
(3) Heidelberg (2007–2011)	11 (11♀): 9 IHCA, 1 H-HCA, 1 with IHCA + H-HCA		34			
(4) London Kings (1998–2011)	35 (30♀): 18 IHCA, 7 H-HCA, 1 *β*-HCA, 9 UHCA					
(5) NY (Mt Sinai) (2007–2011)*	27: 9 H-HCA, 11 I-HCA, 7 UHCA		15			
(6) San Francisco (selected cases)	12 (10♀): 2 IHCA, 3 H-HCA (1 with HCC, 1 borderline), 3 *β*-HCA (2 with HCC, 1 borderline), 4 UHCA (1 with HCC)					
(7) Seattle (2008–2011)	9 (7♀): 3 IHCA, 3 H-HCA, 1 *β*-HCA, 2 UHCA		1		1	
(8) Seoul (2008–2011)	2 (1♀): 1 *β*-IHCA, 1 *β*-HCA (1 with HCC)		4 (3♀)			
(9) Singapore (selected cases)	2 (2♀): 1 *β*-IHCA (with HCC), 1 H-HCA					
(10) Taiwan	12 (5♀): 3 IHCA, 2 *β*-HCA, 1 H-HCA, 6 UHCA					

*See papers in this issue for additional information.

**Table:** (a) LFABP

	T	NT
H-HCA	− (a)	+ (b)
*β*-HCA, IHCA, *β*-IHCA, UHCA	+	+
FNH	+	+
MNR/MNR-FNH-like (cirrhosis)	+	+

(a) Some hepatocytes may be positive at the periphery of the nodule, as well as in between 2 coalescent nodules.

(b) When the expression is weak, reading may be difficult.

**Table:** (b) CRP

	T	NT
IHCA, *β*-IHCA	+ (a)	− (b)
H-HCA, *β*-HCA, UHCA	−	− (b)
FNH	**−** (b^'^)	− (b)
MRN/MRN-HNF-like (cirrhosis)	(c)	(d)

(a) Staining can be heterogeneous.

(b) Can be positive (in case of hemorrhage/necrosis, inflammatory syndrome; portal branch embolization, etc.).

(b^'^) Can be positive if (b) is positive.

(c) Often positive (weak/mild).

(d) Negative to positive (weak to mild), heterogeneous from nodule to nodule.

**Table:** (c) GS

	T	NT
FNH	+ (a)	(b)
MRN/MRN FNH-like (cirrhosis)	(c)	(d)
*β*-HCA, *β*-IHCA	+ (e)	(b)
H-HCA, IHCA, UHCA	−	(b)

(a) Map-like pattern.

(b) Normal positivity around central veins (1–3 rows).

(c) From absence to positivity (limited to veins and/or border of fibrous axis).

(d) Negative, occasional faint staining.

(e) Strong and diffuse or heterogeneous.
