# Supplementary material for: Focal Nodular Hyperplasia and Hepatocellular Adenoma around the World Viewed through the Scope of the Immunopathological Classification
Source: Int J Hepatol. 2013 Apr 14;2013:268625. doi: 10.1155/2013/268625 (PMC3654480; doi:10.1155/2013/268625)
Supplement: Supplementary file 1 — Supplementary Table 1: main publications from European centers dealing with hepatocellular nodules. [file 268625.f1.pdf]

Supplemental table 1 Major references from european centres dealing with hepatocellular nodules (not quoted in the text)

Bordeaux

1. Bioulac-Sage P, Laumonier H, Cubel G, Rossi JZ, Balabaud C. Hepatic resection for inflammatory hepatocellular adenomas: pathological identification of micronodules expressing inflammatory proteins. Liver Int. 2010 ;30:149-54.

2. Laumonier H, Rullier A, Saric J, Balabaud C, Bioulac-Sage P. Unexpected discovery of 2 cases of hepatocyte nuclear factor 1alpha-mutated infracentimetic adenomatosis. World J Gastroenterol. 2008 ;14:4830-3.

3. Sa Cunha A, Blanc JF, Lazaro E, Mellottee L, Le Bail B, Zucman-Rossi J, Balabaud C, Bioulac-Sage P. Inflammatory syndrome with liver adenomatosis: the beneficial effects of surgical management. Gut. 2007 ;56:307-9.

4. Bacq Y, Jacquemin E, Balabaud C, Jeannot E, Scotto B, Branchereau S, Laurent C, Bourlier P, Pariente D, de Muret A, Fabre M, Bioulac-Sage P, Zucman-Rossi J. Familial liver adenomatosis associated with hepatocyte nuclear factor 1alpha inactivation. Gastroenterology. 2003 ;125:1470-5.

5. Lepreux S, Laurent C, Balabaud C, Bioulac-Sage P. FNH-like nodules: Possible precursor lesions in patients with focal nodular hyperplasia (FNH). Comp Hepatol. 2003 ;2:7.

6. Lepreux S, Laurent C, Blanc JF, Trillaud H, Le Bail B, Trouette H, Saric J, Zucman-Rossi J, Balabaud C, Bioulac-Sage P. The identification of small nodules in liver adenomatosis. J Hepatol. 2003 ;39:77-85.

Beaujon

1- Paradis V. Benign liver tumors: an update. Clin Liver Dis. 2010;14:719-29.

2- Ozenne V, Paradis V, Vullierme MP, Vilgrain V, Leblanc T, Belghiti J, Imbert A, Valla DC, Degos F. Liver tumours in patients with Fanconi anaemia: a report of three cases. Eur J Gastroenterol Hepatol. 2008 ;20:1036-9.

3- Paradis V, Benzekri A, Dargère D, Bièche I, Laurendeau I, Vilgrain V, Belghiti

J, Vidaud M, Degott C, Bedossa P. Telangiectatic focal nodular hyperplasia: a

variant of hepatocellular adenoma. Gastroenterology. 2004 ;126:1323-9..

Créteil

1-Luciani A, Kobeiter H, Maison P, Cherqui D, Zafrani ES, Dhumeaux D, Mathieu D. Focal nodular hyperplasia of the liver in men: is presentation the same in men and women? Gut. 2002 ;50:877-80.

2-Mathieu D, Kobeiter H, Maison P, Rahmouni A, Cherqui D, Zafrani ES, Dhumeaux D. Oral contraceptive use and focal nodular hyperplasia of the liver. Gastroenterology. 2000;118:560-

3-Mathieu D, Kobeiter H, Cherqui D, Rahmouni A, Dhumeaux D. Oral contraceptive intake in women with focal nodular hyperplasia of the liver. Lancet. 1998 ;352:1679-80.

4-Cherqui D, Rahmouni A, Charlotte F, Boulahdour H, Métreau JM, Meignan M, Fagniez PL, Zafrani ES, Mathieu D, Dhumeaux D. Management of focal nodular hyperplasia and hepatocellular adenoma in young women: a series of 41 patients with clinical, radiological, and pathological correlations. Hepatology. 1995 ;22:1674-81. P

5-Bryant R, Laurent A, Tayar C, Cherqui D. Laparoscopic liver resection-understanding its role in current practice: the Henri Mondor Hospital experience. Ann Surg. 2009 ;250:103-11. P

6-Mathieu D, Zafrani ES, Anglade MC, Dhumeaux D. Association of focal nodular hyperplasia and hepatic hemangioma. Gastroenterology. 1989 ;97:154-7.

7-Golli M, Van Nhieu JT, Mathieu D, Zafrani ES, Cherqui D, Dhumeaux D, Vasile N, Rahmouni A. Hepatocellular adenoma: color Doppler US and pathologic correlations. Radiology. 1994 190:741-4.

8-Cherqui D, Rahmouni A, Charlotte F, Boulahdour H, Métreau JM, Meignan M, Fagniez PL, Zafrani ES, Mathieu D, Dhumeaux D. Management of focal nodular hyperplasia and hepatocellular adenoma in young women: a series of 41 patients with clinical, radiological, and pathological correlations. Hepatology. 1995;22:1674-81.

Rotterdam

1-van Vledder MG, van Aalten SM, Terkivatan T, de Man RA, Leertouwer T, Ijzermans JN. Safety and efficacy of radiofrequency ablation for hepatocellular adenoma. J Vasc Interv Radiol. 2011 ;22:787-93.

2-van Aalten SM, Terkivatan T, van der Linden E, Verheij J, de Man RA, Ijzermans JN. Management of liver adenomatosis by radiofrequency ablation. Dig Surg. 2011;28:173-7.

3- van Aalten SM, Verheij J, Terkivatan T, Dwarkasing RS, de Man RA, Ijzermans JN. Validation of a liver adenoma classification system in a tertiary referral centre: implications for clinical practice. J Hepatol. 2011 ;55:120-5

4-Noels JE, van Aalten SM, van der Windt DJ, Kok NF, de Man RA, Terkivatan T, Ijzermans JN. Management of hepatocellular adenoma during pregnancy. J Hepatol. 2011 ;54:553-8.

5-van Aalten SM, Terkivatan T, de Man RA, van der Windt DJ, Kok NF, Dwarkasing R, Ijzermans JN. Diagnosis and treatment of hepatocellular adenoma in the Netherlands: similarities and differences. Dig Surg. 2010;27:61-7.

6-van der Sluis FJ, Bosch JL, Terkivatan T, de Man RA, Ijzermans JN, Hunink MG. Hepatocellular adenoma: cost-effectiveness of different treatment strategies. Radiology. 2009 ;252:737-46.

7-Furlan A, van der Windt DJ, Nalesnik MA, Sholosh B, Ngan KK, Pealer KM, Ijzermans JN, Federle MP. Multiple hepatic adenomas associated with liver steatosis at CT and MRI: a case-control study. AJR Am J Roentgenol. 2008 ;191:1430-5.

8-van der Windt DJ, Kok NF, Hussain SM, Zondervan PE, Alwayn IP, de Man RA, IJzermans JN. Case-orientated approach to the management of hepatocellular adenoma. Br J Surg. 2006 ;93:1495-502.

9-Terkivatan T, van den Bos IC, Hussain SM, Wielopolski PA, de Man RA,IJzermans JN. Focal nodular hyperplasia: lesion characteristics onstate-of-the-art MRI including dynamic gadolinium-enhanced and superparamagnetic iron-oxide-uptake sequences in a prospective study. J Magn Reson Imaging. 2006 ;24:864-72. Pub

Amsterdam

1-Lin H, van den Esschert J, Liu C, van Gulik TM. Systematic review ofhepatocellular adenoma in China and other regions. J Gastroenterol Hepatol. 2011 ;26:28-35.

2-van den Esschert JW, Bieze M, Beuers UH, van Gulik TM, Bennink RJ.Differentiation of hepatocellular adenoma and focal nodular hyperplasia using 18F-fluorocholine PET/CT. Eur J Nucl Med Mol Imaging. 2011 ;38:436-40.

3-van den Esschert JW, van Gulik TM, Phoa SS. Imaging modalities for focal nodular hyperplasia and hepatocellular adenoma. Dig Surg. 2010;27:46-55.

4-van Gulik TM, van den Esschert JW, Erdogan D, Vetelaïnen R. Hepatocellular adenomas. Liver Int. 2009 ;29:616.

5-Veteläinen R, Erdogan D, de Graaf W, ten Kate F, Jansen PL, Gouma DJ, van Gulik TM. Liver adenomatosis: re-evaluation of aetiology and management. Liver Int. 2008 ;28:499-508.

London King's

1-Sakellariou S, Al-Hussaini H, Scalori A, Samyn M, Heaton N, Portmann B, Tobal K, Quaglia A. Hepatocellular adenoma in glycogen storage disorder type I: a clinicopathological and molecular study. Histopathology. 2012;60:E58-65. Feb 28. PubMed PMID: 22372484.

2- Sakellariou S, Morgan Y, Heaton N, Portmann B, Quaglia A, Tobal K. New monoallelic (partial tandem duplication) mutation of HNF1a gene in steatotic hepatocellular adenoma. Eur J Gastroenterol Hepatol. 2011 ;23:623-7.

London RFH

1-Ferrell LD, Crawford JM, Dhillon AP, Scheuer PJ, Nakanuma Y. Proposal for standardized criteria for the diagnosis of benign, borderline, and malignant hepatocellular lesions arising in chronic advanced liver disease. Am J Surg Pathol. 1993;17:1113-23.

Brussels

1-Brenard R, Chapaux X, Deltenre P, Henrion J, De Maeght S, Horsmans Y, Borbath I, Leenaerts A, Van Cauter J, Francque S, Sersté T, Moreno C, Orlent H, Mengeot P, Lerut J, Sempoux C. Large spectrum of liver vascular lesions including high prevalence of focal nodular hyperplasia in patients with hereditary haemorrhagic telangiectasia: the Belgian Registry based on 30 patients. Eur J Gastroenterol Hepatol. 2010 ;22:1253-9.

2-Leconte I, Van Beers BE, Lacrosse M, Sempoux C, Jamart J, Materne R, Baudrez V, Horsmans Y. Focal nodular hyperplasia: natural course observed with CT and MRI. J Comput Assist Tomogr. 2000 ;24:61-6.

3-Dardenne S, Hubert C, Sempoux C, Annet L, Jouret-Mourin A, Horsmans Y, Van Beers BE, Zech F, Gigot JF. Conservative and operative management of benign solid hepatic tumours: a successful stratified algorithm. Eur J Gastroenterol Hepatol. 2010 ;22:1337-44.

Leuwen

1-Libbrecht L, Cassiman D, Verslype C, Maleux G, Van Hees D, Pirenne J, Nevens F, Roskams T. Clinicopathological features of focal nodular hyperplasia-like nodules in 130 cirrhotic explant livers. Am J Gastroenterol. 2006;101:2341-6.

2-Vander Borght S, Libbrecht L, Blokzijl H, Faber KN, Moshage H, Aerts R, Van Steenbergen W, Jansen PL, Desmet VJ, Roskams TA. Diagnostic and pathogenetic implications of the expression of hepatic transporters in focal lesions occurring in normal liver. J Pathol. 2005 ;207:471-82.

3-Libbrecht L, De Vos R, Cassiman D, Desmet V, Aerts R, Roskams T. Hepatic progenitor cells in hepatocellular adenomas. Am J Surg Pathol. 2001;25:1388-96.

Brescia

1-Morana G, Grazioli L, Kirchin MA, Bondioni MP, Faccioli N, Guarise A, Schneider G. Solid hypervascular liver lesions: accurate identification of true benign lesions on enhanced dynamic and hepatobiliary phase magnetic resonance imaging after gadobenate dimeglumine administration. Invest Radiol. 2011;46:225-39.

2-Zech CJ, Grazioli L, Breuer J, Reiser MF, Schoenberg SO. Diagnostic performance and description of morphological features of focal nodular hyperplasia in Gd-EOB-DTPA-enhanced liver magnetic resonance imaging: results of a multicenter trial. Invest Radiol. 2008;43:504-11.

3-Grazioli L, Morana G, Kirchin MA, Schneider G. Accurate differentiation of focal nodular hyperplasia from hepatic adenoma at gadobenate dimeglumine-enhanced MR imaging: prospective study. Radiology. 2005;236:166-77.

4-Blachar A, Federle MP, Ferris JV, Lacomis JM, Waltz JS, Armfield DR, Chu G, Almusa O, Grazioli L, Balzano E, Li W. Radiologists' performance in the diagnosis of liver tumors with central scars by using specific CT criteria. Radiology. 2002;223:532-9.

5-Grazioli L, Morana G, Kirchin MA, Caccia P, Romanini L, Bondioni MP, Procacci C, Chiesa A. MRI of focal nodular hyperplasia (FNH) with gadobenate dimeglumine (Gd-BOPTA) and SPIO (ferumoxides): an intra-individual comparison. J Magn Reson Imaging. 2003;17:593-602.

6-Grazioli L, Morana G, Federle MP, Brancatelli G, Testoni M, Kirchin MA, Menni K, Olivetti L, Nicoli N, Procacci C. Focal nodular hyperplasia: morphologic and functional information from MR imaging with gadobenate dimeglumine. Radiology. 2001;221:731-9..

7-Brancatelli G, Federle MP, Grazioli L, Blachar A, Peterson MS, Thaete L. Focal nodular hyperplasia: CT findings with emphasis on multiphasic helical CT in 78 patients. Radiology. 2001;219:61-8..
